# BIDpred: unraveling B cell Immunodominance hierarchical pattern using statistical feature discovery and deep learning prediction

**DOI:** 10.3389/fimmu.2025.1646946

**Published:** 2025-08-13

**Authors:** Sungjin Choi, Dongsup Kim

**Affiliations:** Department of Bio and Brain Engineering, Korea Advanced Institute of Science and Technology, Daejeon, Republic of Korea

**Keywords:** immunoinformatics, B cell immunodominance, vaccine design, deep learning, protein language model

## Abstract

Knowledge of B cell immunodominance is important for designing vaccines that may elicit effective immune responses. However, the prevalence and characteristics of B cell immunodominance remain poorly understood. In this study, we introduced an immunodominance score through novel data processing methods and identified statistically significant characteristics of B cell immunodominance at the residue and patch levels. Based on these findings, we developed BIDpred, a B cell ImmunoDominance predictor, that learns newly discovered features by leveraging protein language model embeddings and graph attention network to predict the immunodominance scores. BIDpred demonstrates superior performance in predicting immunodominance scores compared to existing methods while maintaining competitive accuracy with state-of-the-art methods for conventional B cell epitope prediction. To the best of our knowledge, this is the first study to systematically analyze and predict B cell immunodominance patterns, marking a significant advancement in vaccine design research.

## Introduction

Immunodominance (ID), described as a hierarchical level of preference for immune response in antigen, is essential for understanding adaptive immunity. In vaccine design, ID is often used to elicit the intended immune response in epitope-based vaccines (1). While T cell ID has been extensively studied (2–4), relatively little is known about B cell ID (5). Furthermore, even the existence of B cell epitopes has been argued that epitopes could be everywhere on the surface of antigens (6). Despite this ambiguity, structural studies of antibody-antigen complexes, such as those involving influenza A and SARS-CoV-2, reveal certain levels of immunological preference among antigen amino acid residues; some residues of antigens appear more likely to bind antibodies (7, 8).

Numerous computational approaches have been developed to predict B cell epitopes for vaccine design applications (9–21). However, previous methods have overlooked the hierarchical nature of ID between epitopes as they were solely trained on the binary labels by classifying residues as either epitopes or non-epitopes. These labels were typically defined using distance-based annotations derived from antigen-antibody complex structures. We conjecture that incorporating ID scores, estimated using a novel data processing strategy, could provide additional insights into the relative immunological preference of certain epitopes over others. ID scores are continuous scores ranging from 0 to 1, which represent the epitope priorities against the antibody interaction. Although the ID score defined in this study may not perfectly capture the biological phenomenon due to limited data availability, it could still serve as a reasonable approximation to enhance our understanding of epitope prioritization.

We defined the ID score through a comprehensive data curation process, which involves sequence clustering, multiple sequence alignment (MSA) building, and epitope annotation. Statistical analyses were then conducted to investigate the physico-chemical, geometrical, evolutionary, and compositional characteristics associated with B cell ID. Residue- and patch-level analyses revealed statistically significant correlations with several features, particularly strong signals related to conservation and clustering patterns.

Building on these insights, we developed BIDpred, a predictive model that leverages highly significant features identified in the statistical analysis. BIDpred integrates pretrained protein language model embeddings with a graph attention network (GAT) to capture the nuanced features of B cell ID. BIDpred demonstrates superior performance in predicting immunodominance scores compared to existing methods while maintaining comparable accuracy with the state-of-the-art methods for conventional B cell epitope prediction. This highlights the value of our novel data processing approach in providing additional insights into immune response preferences among epitopes. Code and dataset are available at https://github.com/sj584/BIDpred and webserver at http://bidpred.kaist.ac.kr.

## Materials and methods

### Data curation

**Figure 1:**
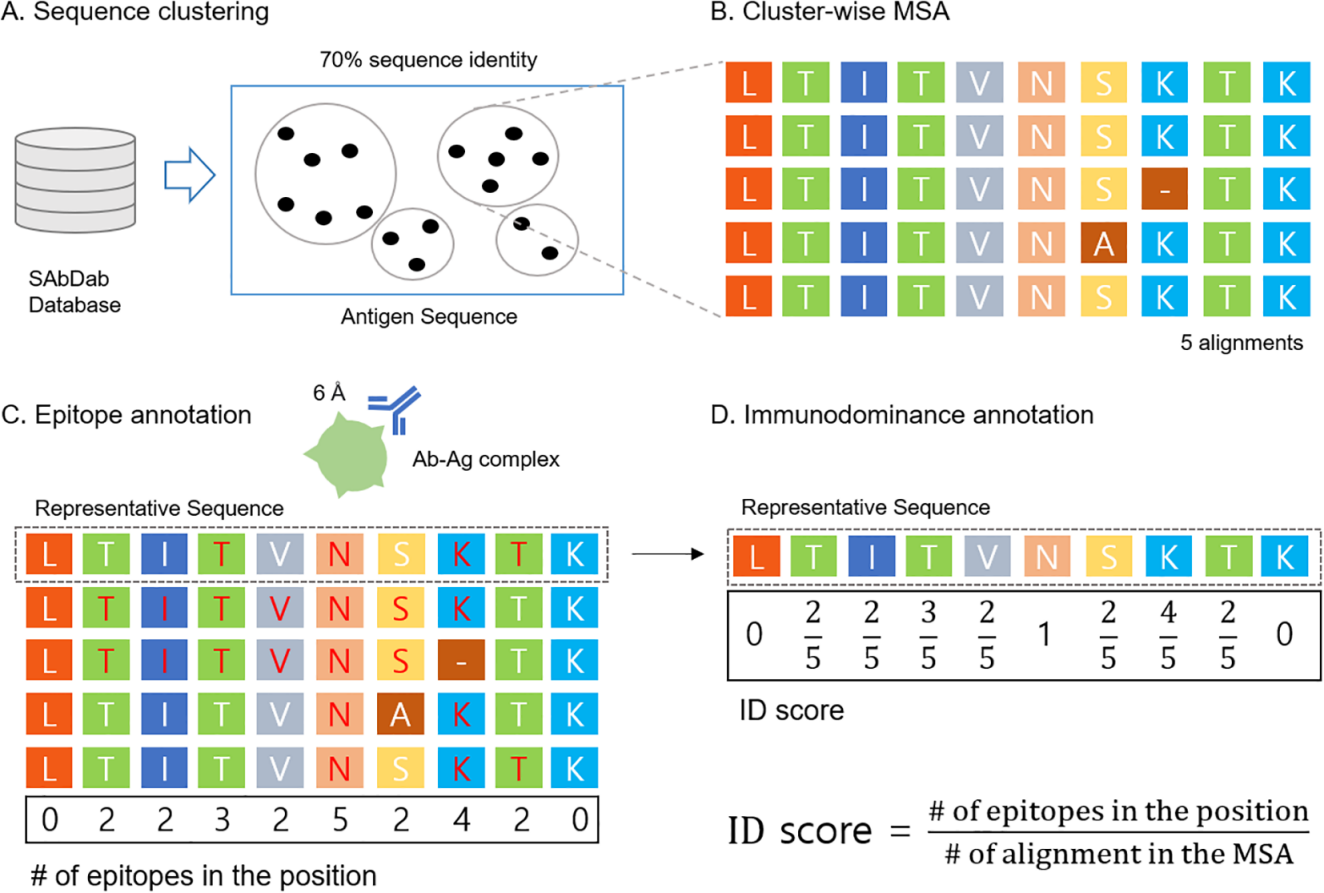
Data curation process **(A)** Antibody-antigen structural dataset was downloaded from SAbDab and the antigen sequence was clustered using mmseq2 with 70% sequence identity. **(B)** MSA was built using ClustalW for each cluster. **(C)** Epitopes were annotated based on the antibody-antigen structure (6Å cutoff) and then mapped to each sequence in the MSA (residues in red color). **(D)** B cell ID scores were calculated by the ID score definition and mapped to the representative sequence.

X-ray crystallography data were collected from SAbDab (22) as of Mar.19, 2024. The data curation process is illustrated in **Figure 1**. For quality filtering, we used a filtering cutoff of resolution 3.0Å, R-factor 0.25, antigen size with at least 50 amino acids, and maximum antibody sequence identity of 99% (23). After quality filtering, we extracted the antigen sequences and performed sequence clustering using the mmseq2 *easy-cluster* command with a minimum sequence identity threshold of 0.70; all other options were set to default (24) (**Figure 1A**). Clusters with at least 4 elements were used. One cluster can be viewed as a group of the same antigens interacting with different antibodies. Within each cluster, MSAs were constructed using the Clustal Omega *clustalo* command with default settings (25) (**Figure 1B**). Further information about the MSA depth for each cluster can be found in **Supplementary Table S1**. In each MSA, we annotated epitopes from the antibody-antigen complex structures. Antigen residues within 6Å distance to antibody residues were defined as epitopes. After epitope annotation was mapped to each sequence in MSA (**Figure 1C**), ID scores were assigned to the representative sequence of the MSA (**Figure 1D**). In summary, each data point corresponds to a representative protein from its own cluster, with the ID score annotated based on epitope annotation in cluster-wise MSA.

Motivated by the previous study (26), ID score is defined by,


$$ID\;score=\;\frac{\#\;of\;epitopes\;in\;the\;position}{\#\;of\;alignment\;in\;the\;MSA}$$

Since the number of epitopes at a given position in an MSA is always less than or equal to the number of alignments in the MSA, ID score could only range from 0 to 1. For the data split, we reasoned that the representative sequence with more alignments in the MSA would better reflect the characteristics of B cell ID. Consequently, we split the data such that the test set included representative sequences with at least 10 alignments in the MSA, while the training sets contained representative sequences with 4 to 9 alignments in the MSA. This resulted in a total of 92 training sets and 24 test sets. The distribution of the antigen type can be found in **Supplementary Figure S1** and a full list of antigen types is provided in **Supplementary Table S11**. Other characteristics such as antigen size, epitope, non-epitope, and immunodominance can be found in **Supplementary Figure S2**.

### Feature embeddings

A molecular graph was generated from the protein structure. Nodes were amino acid residues, and edges were constructed when nodes were close to each other within 10Å distance. ESM-based residue representation was used for node embedding. For ESM-2 (27), *esm2_t33_650M_UR50D* model was used. For ESM-IF1 (28), *esm_if1_gvp4_t16_142M_UR50* model was used.

### Statistical analysis of protein features

Statistical analysis was performed on the representative sequences in the test set, which has representative sequences with at least 10 alignments in MSA. We used an independent sample T-test for amino acid feature analysis, while a Mann-Whitney U-test was used for compositional analysis. The null hypothesis was rejected at a significance level of α≤0.05 . Afterwards, we performed Benjamini-Hochberg procedure for multiple testing correction. Geometric features, physico-chemical features, compositional (either amino acid or secondary structure), and evolutionary features were examined at both residue and patch levels (**Supplementary Table S2**). Surface residues with at least relative surface accessibility (RSA) 0.10 were used. Surface patches were constructed by grouping surface residues within a 10 Å distance of central surface residues.

### Features for statistical analysis

RSA and secondary structure were collected from DSSP (29) module in Biopython (30). Protrusion and residue depth were obtained from PSAIA (31). Hydrophobicity, isoelectric point, residue volume, steric, polarizability, H-bond donor, polarity, positive charge, and negative charge were acquired from AAIndex (32) (**Supplementary Table S3**). Per**-**residue conservation score was obtained from ConSurf (33).

### Hyperparameters

Hyperparameters were optimized to achieve the lowest validation loss. Specifically, 200 epochs, batch size of 4, 8 multi-attention heads, learning rate of 1e-6, Adam optimizer, mean squared error loss, 3 GAT layers with hidden dimensions of 2048-512-128, and 2 fully connected (FC) layers with dimensions of 128-32–1 were used.

### Evaluation metrics

B cell ID is a hierarchical level of preference in immune response. Therefore, Spearman correlation was the main criterion for measuring the ID. Also, R-squared (*R* ^2^) score and Pearson correlation were used for additional evaluation. For B cell epitope prediction, area under the receiver operating characteristic curve (AUC-ROC) and area under the precision-recall curve (AUC-PR) were used for threshold independent evaluation for classification.

## Result

### Statistical analysis reveals B cell ID in residue and patch level

To investigate which characteristics are related to the B cell immunodominant regions, statistical analysis was performed to examine geometrical, physicochemical, compositional, and evolutionary features at both the residue and patch levels (**Supplementary Table S2**). Only surface residues with a relative surface accessibility (RSA) of at least 0.10 were considered. Surface patches were generated from the central node with the same surface criteria. We defined the two ID groups based on the following criteria;


$$\left\{ \begin{array}{c} highly\;immunodominant,\;if\;ID\geq 0.5*\max immunodominance\;of\;protein\\ \;\;weakly\;immunodominant,\;if0<ID<0.2*\max immunodominance\;of\;protein \end{array}\right.$$

**Figure 2:**
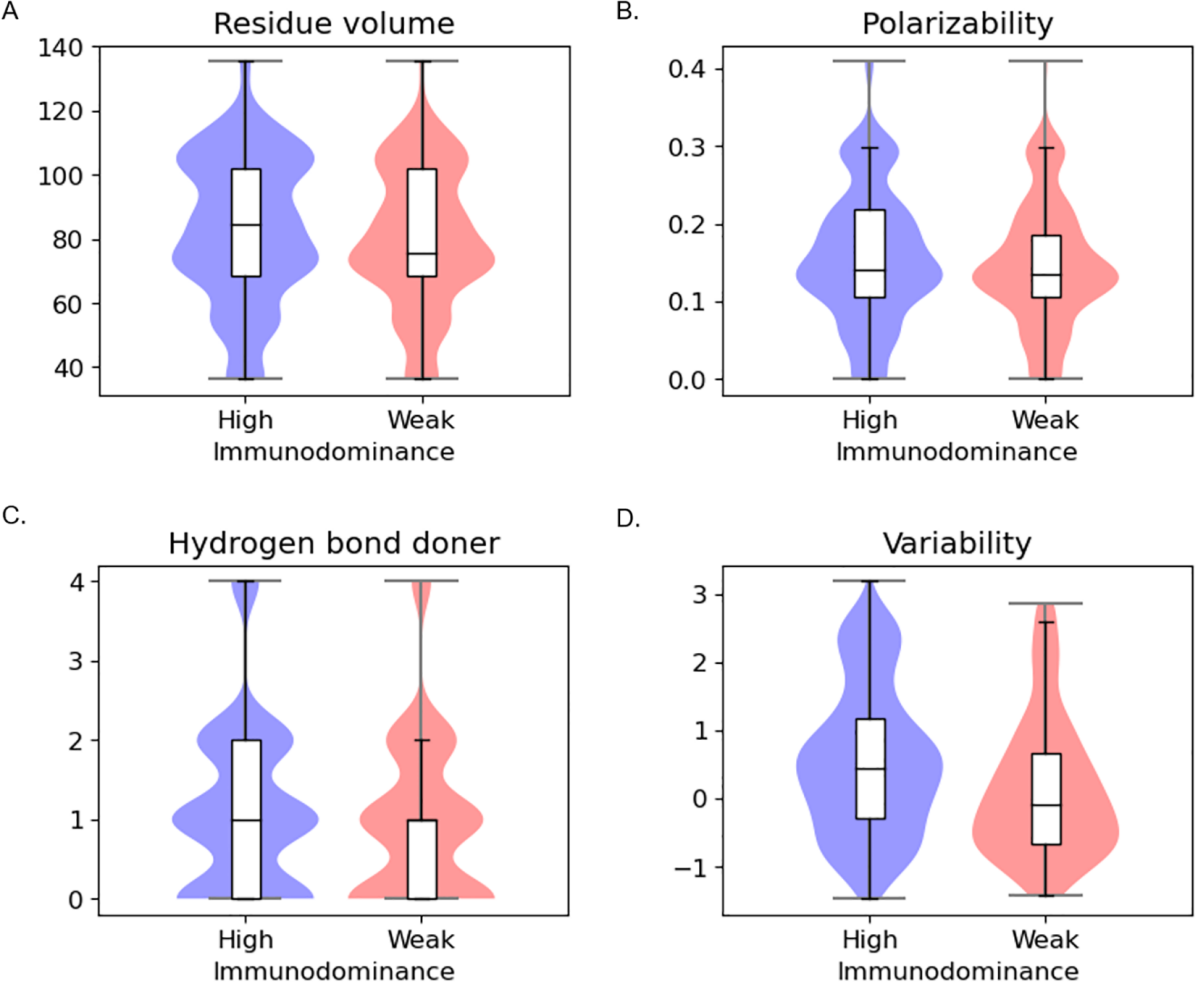
Residue-level statistical analysis between high immunodominance and weak immunodominance groups **(A)** Residue volume **(B)** Polarizability **(C)** Hydrogen bond donor **(D)** Conservation.

In residue-wise analysis (**Figure 2**, **Supplementary Table S4**), highly immunodominant residues exhibit distinct patterns in several features, including residue volume, polarizability, hydrogen bond donor, and conservation. Specifically, highly immunodominant residues tend to have bigger residue volume, attractive interactions mediated by electrons, act as stronger hydrogen bond donors, and display greater variability in sequence conservation.

**Figure 3:**
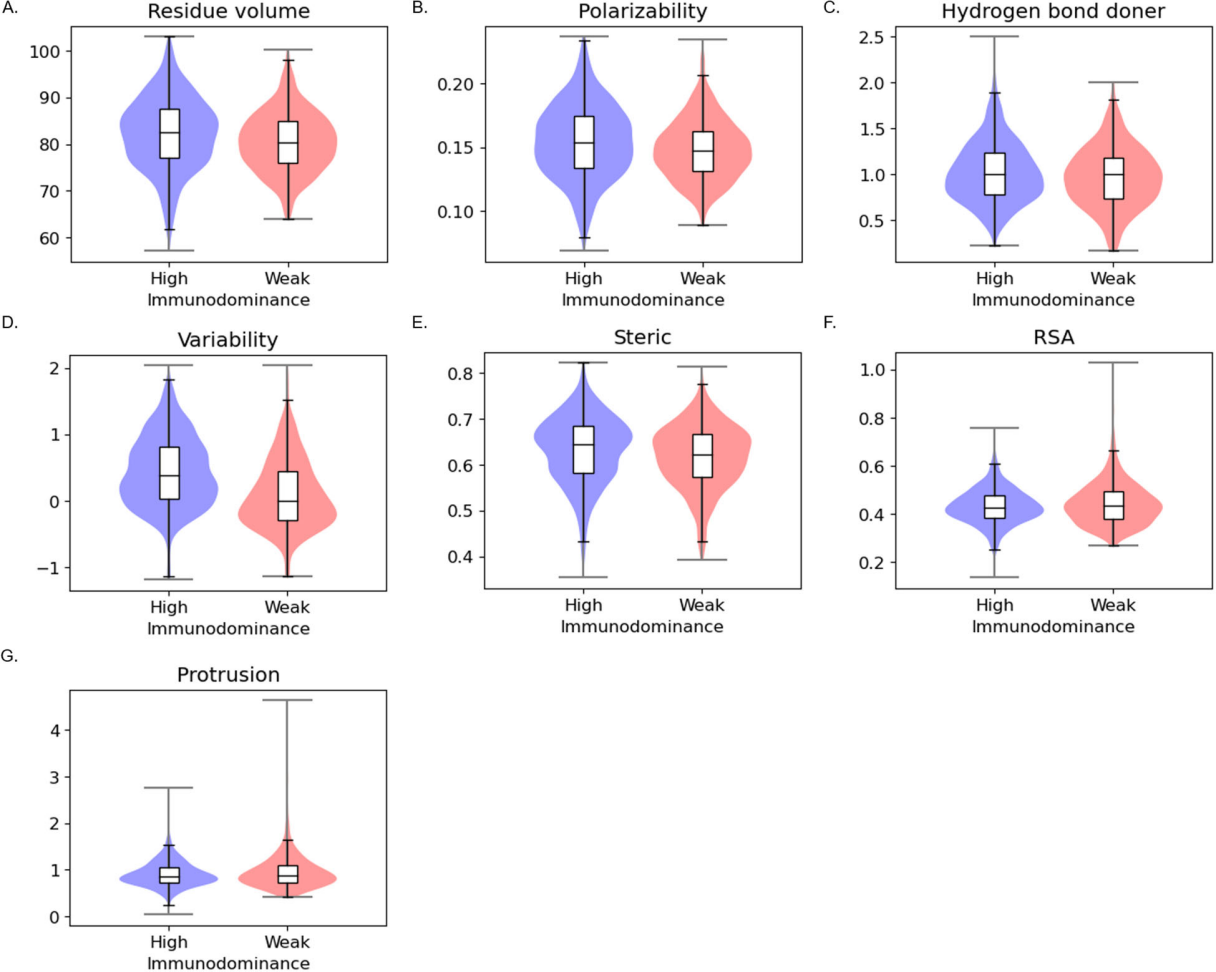
Patch-level statistical analysis between the high immunodominant and weak immunodominant groups **(A)** Residue volume **(B)** Polarizability **(C)** Hydrogen bond donor **(D)** Conservation **(E)** Steric **(F)** RSA **(G)** Protrusion.

In patch-level analysis (**Figure 3**, **Supplementary Table S4**), we observed that features identified in residue-level analysis are still valid, implying that amino acids with similar properties are located close to each other. We additionally found different patterns in steric, RSA, and protrusion with statistical significance. In the patch level, highly immunodominant patches were additionally found to be highly steric, less exposed, and less protruding.

**Figure 4:**
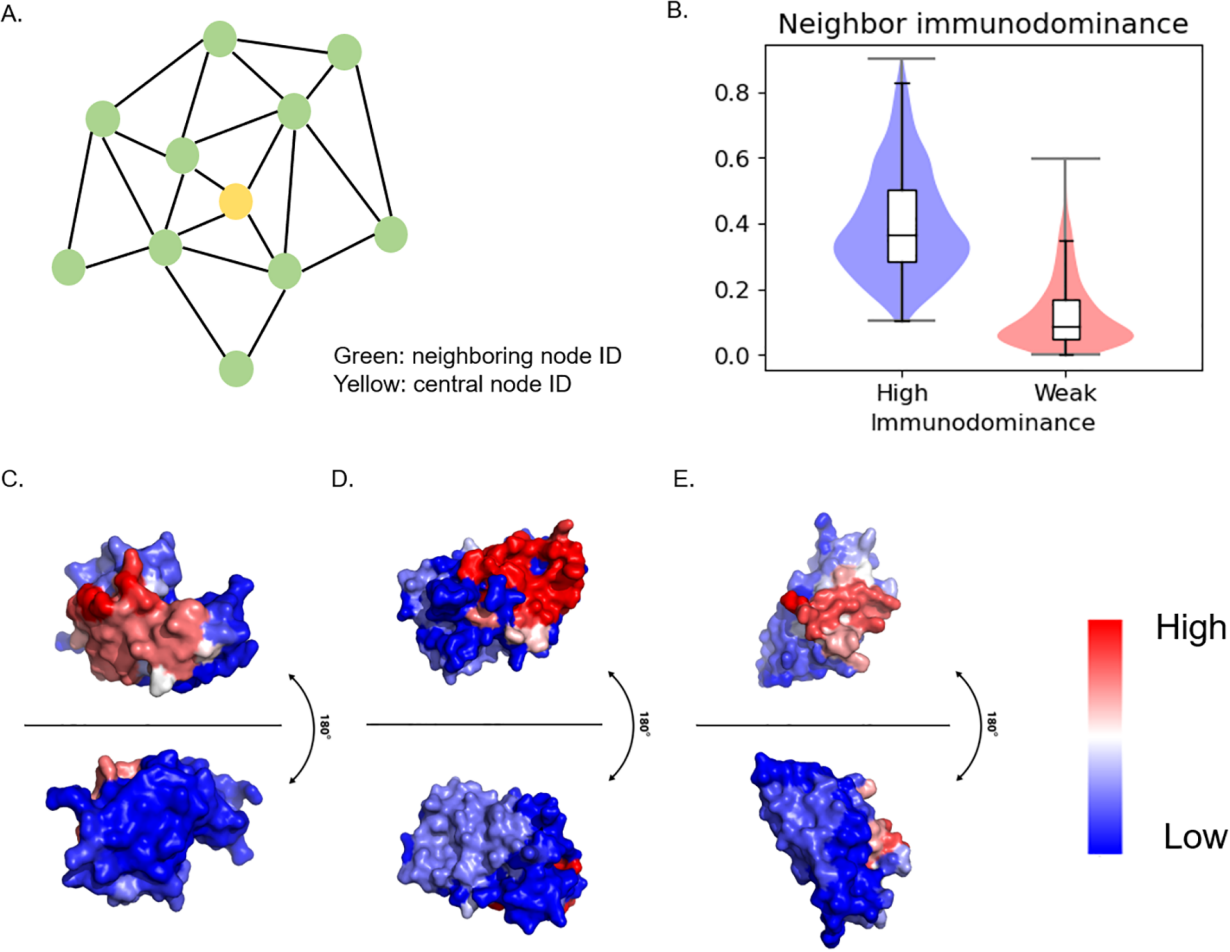
Neighboring immunodominance analysis and visualization **(A)** Schematics of Surface patch. Patch was generated from 10 Å distance from the central node. Patch ID was defined by central node ID. Neighbor ID was the average value of the neighboring node ID score. **(B)** Statistical analysis of neighbor immunodominance. **(C)** PDB ID: 1FBI, X chain **(D)** PDB ID: 4YPG, D chain **(E)** PDB ID: 8JEL, J chain.

Based on the observation that similar residues are close to each other in patch analysis, we investigated the distribution of immunodominant residues within a patch by analyzing neighbor immunodominance. For this analysis, residue ID scores in the patch were averaged while excluding the central node ID score (**Figure 4A**). Here, a drastic difference pattern was observed (**Figure 4B**, **Supplementary Table S4**). The result demonstrates immunodominant residues tend to cluster closely together. This clustering pattern was further visualized using PyMol (34), which clearly highlights the spatial grouping of immunodominant residues in B cell ID (**Figures 4C–E**).

### B cell ID prediction task

**Figure 5:**
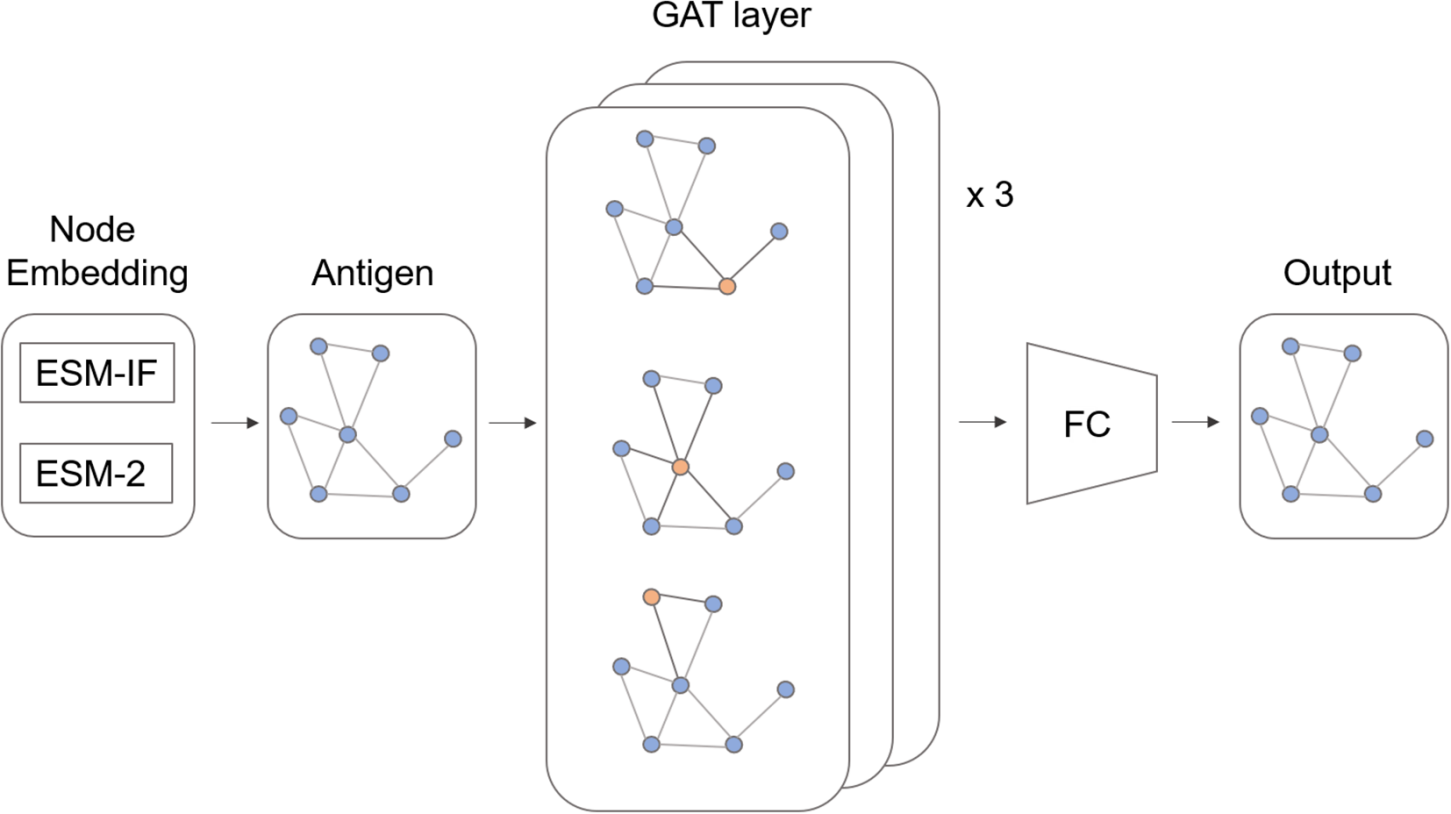
Model architecture. ESM-based pretrained model embeddings were used for node features. The antigen structure was represented as a molecular graph and encoded by graph attention (GAT) layer. A fully connected (FC) layer predicts the immunodominance score.

Thus far, we have identified several features associated with B cell immunodominance (ID). With the aforementioned features in mind, we further explored B cell ID prediction methods using deep learning. However, most prediction models based on statistical features failed to achieve satisfactory performance, likely due to weak signals that were insufficiently detectable. Meanwhile, we noted that conservation and ID clustering patterns showed strong statistical significance with low p-value (**Supplementary Table S4**). Thus, we devised a model which leverages evolutionary and clustering features in an efficient way (**Figure 5**). We used ESM-2 (27) sequence embedding and ESM-IF1 (28) structure embedding to capture the evolutionary features of the B cell ID. To capture the structural homophily of the B cell ID, we used graph attention network which uses message passing node updates from the adjacent nodes.

We evaluated our model on B cell ID benchmark datasets and compared it with existing B cell epitope prediction methods (**Table 1**). The main criterion is Spearman correlation coefficient, which measures the hierarchical preference of the immunogenic residues. For this analysis, redundancy was removed by ensuring 70% sequence identity between each test set and the training sets of the B epitope predictors. Since each model has a different training set, subset of our test set was used for evaluation in comparison with our model. This resulted in four separate evaluations. Across 24 test sets, our model consistently achieved the highest Spearman correlation scores, outperforming all other models. While ElliPro, SEPPA3, and CBTope showed almost random performance in ID prediction, BepiPred-3 and DiscoTope-3 were reported to have certain levels of capturing immunodominance (**Supplementary Table S5**). We reasoned that those models, being trained on ESM-based evolutionary features with their redundant training sets, might have partially learned ID patterns. Evaluation results for all models using independent and the same test set are provided in **Supplementary Table S6**.

**Table 1:** B cell ID benchmark in comparison to conventional B cell epitope predictors.

Method	Pearson	Spearman	R2 score	# of PDB test
ElliPro	0.078	0.057	-5.165	
Ours	**0.393**	**0.406**	**0.144**	24/24
SEPPA3	0.088	-0.015	-0.096	
Ours	**0.422**	**0.396**	**0.169**	14/24
CBTope	0.065	0.017	-1.250	
Ours	**0.374**	**0.346**	**0.136**	20/24
Bepipred-3	0.338	0.437	0.096	
DiscoTope-3	0.405	0.444	0.018	
Ours	**0.429**	**0.476**	**0.181**	6/24

Bold values indicate the highest score in the benchmark.

### BIDpred method showed comparable results in conventional B cell epitope prediction

We also compared our model with other methods in the conventional B cell epitope prediction task (**Table 2**). We used Epitope3D (35) benchmark dataset with 45 PDB. Again, the redundancy in the test set was removed by applying a 70% sequence identity threshold. In this evaluation, our model demonstrated results comparable to those of current state-of-the-art models. Notably, this performance was achieved despite our model being exclusively trained on the B cell ID prediction task. This outcome supports our initial hypothesis that the B cell ID prediction task captures essential features relevant to B cell epitope prediction.

**Table 2:** B cell epitope prediction benchmark from the epitope3D external dataset.

Method	AUC	AUC-PR	MCC
SEPPA 3	0.55	0.11	-0.02
ElliPro	0.48	0.09	-0.02
epitope3D	0.49	0.08	-0.03
CBTope	0.55	0.13	0.01
Bepipred-3.0	0.71	0.20	0.08
DiscoTope-3.0	0.71	0.19	0.09
Ours	0.71	0.21	0.15

### Ablation study

We conducted an ablation study to evaluate the impact of the features used in our ID prediction model (**Table 3**, **Supplementary Table S7**). The study revealed that both ESM features outperformed the conventional one-hot encoding method, commonly used as a feature representation. Among the two, ESM-IF1 (structural evolutionary features) proved to be more effective than ESM-2 (sequential evolutionary features). The best performance was achieved when both features were combined, highlighting their complementary nature in capturing B cell ID characteristics. The superior importance of structural evolutionary features can be attributed to the spatial clustering of highly immunodominant residues in 3D space, emphasizing the role of structural context in B cell ID learning.

**Table 3:** Ablation study on ID prediction test set.

Ablation	Pearson	Spearman	R2 score
One-hot	-0.027	-0.006	-0.052
ESM-2	0.328	0.302	0.097
ESM-IF1	0.367	0.368	0.123
Ours	**0.393**	**0.406**	**0.144**

Bold values indicate the highest score in the benchmark.

### Case study: SARS-CoV-2

**Figure 6:**
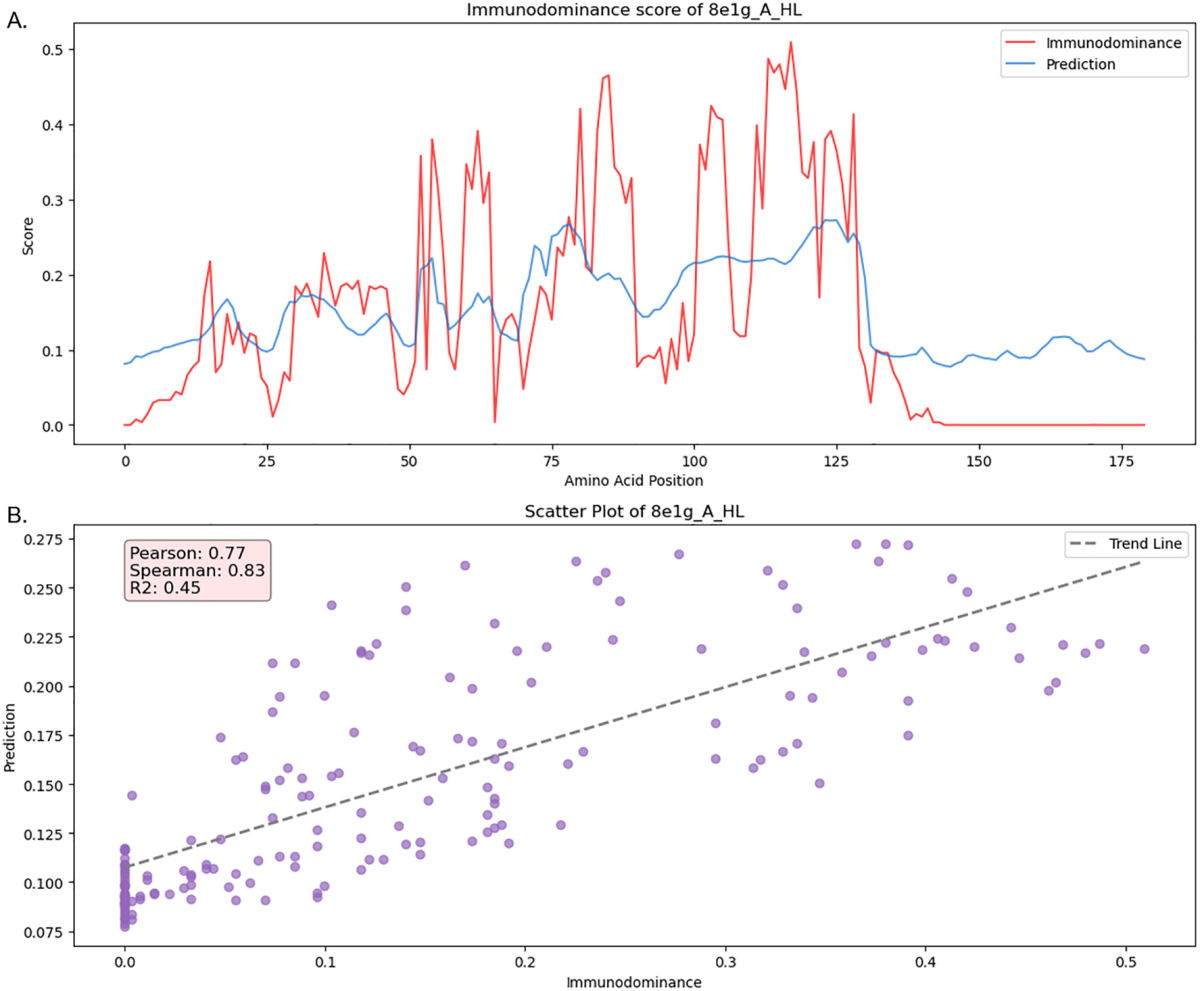
Case study of SARS-CoV-2 immunodominance pattern and model prediction from test set (PDB ID: 8e1g) **(A)** Immunodominance/prediction score per amino acid position **(B)** Scatter plot of prediction and immunodominance with evalution metrics.

We performed a case study on SARS-CoV-2 in the test set (**Figure 6**), which included a large number of structures, resulting in the most extensive MSA alignments (271 alignments). In **Figure 6A**, we visualized the ID pattern and prediction results along the residue positions. The model predictions closely aligned with the SARS-CoV-2 ID pattern, accurately identifying ID peaks in most cases. This agreement was reflected in strong Spearman, Pearson, and *R* ^2^ scores. Additionally, the scatter plot shown in **Figure 6B** illustrates a clear linear relationship between the model predictions and the actual ID pattern, further validating the model’s predictive accuracy for SARS-CoV-2. In comparison with other previous tools, our model demonstrated the best performance, as shown in **Supplementary Table S8**.

## Discussion

B cell ID remains relatively unexplored from statistical and computational perspectives, despite its biological significance. For example, in the influenza A virus, the hemagglutinin (HA) head is typically the primary target of immune responses, whereas the HA stem is a subdominant region. However, the HA head is highly mutable, which often renders previous vaccines ineffective against new variants. To address this challenge, vaccine development efforts are focusing on targeting the less immunodominant but more conserved HA stem, aiming to create universal vaccines (36, 37). In our dataset, influenza A virus ID scores are high on the HA head instead of the stem. Also, the statistical analysis on variability aligns with the conservation pattern in Influenza A virus, which suggests that our data processing well approximates the biological pattern. For many pathogens with unclear ID patterns due to limited data, ID prediction tools can serve as a valuable resource for vaccine discovery, facilitating the identification of conserved and strategically targetable regions.

In summary, we systematically investigated B cell immunodominance (ID) through statistical analysis and developed a prediction model tailored to this task. We curated a dataset specifically designed to capture B cell ID using a variety of bioinformatics tools. Our analysis identified key characteristics associated with B cell ID, highlighting statistically significant features. After statistically identifying the B cell ID, we developed a deep learning method, BIDpred, specifically trained on the B cell ID dataset. We proposed that the conventional B cell epitope prediction task closely aligns with B cell ID prediction task, but existing models were not optimally trained for the B cell ID prediction. BIDpred demonstrated the ability to perform both tasks effectively by capturing the essence of antibody-agnostic B cell epitope prediction. To our knowledge, this work represents the first attempt to statistically analyze B cell ID and to train a deep learning model optimally for B cell ID prediction, paving the way for advancements in vaccine design and immunological research.

## Conclusion

B cell ID is crucial for vaccine design. However, few studies about B cell ID were available. In this study, we introduced a B cell ID score, conducted a comprehensive statistical analysis of B cell ID features, and developed a deep learning-based prediction model. Our findings and predictive tools have the potential to accelerate vaccine development and stimulate further research into B cell ID, enhancing our understanding of adaptive immunity.

The limitation of this work is the deficiency of the data being clustered, which might lead to incomplete B cell ID annotation. Each data point is a representative protein of all proteins in a single cluster. While the representative proteins in the test set were selected to have at least 10 alignments in the MSA, those in the training set had fewer alignments, ranging from 4 to 9. This discrepancy suggests that the training set may not fully capture the robustness of the test set, potentially affecting model performance. In **Supplementary Table S9**, we present results from random shuffling of the train and test sets. When the test set was randomly selected, it often contained very few representative proteins with high MSA depth, resulting in noised training and test outcomes. However, when we ensured that the test set included at least half of its representative proteins with high MSA depth, model training and testing became more stable. This suggests that the rigorous evaluation of model predictions requires a test set with a sufficient number of representative proteins with high MSA depth. As more antigen-antibody structural data becomes available, the dataset could better approximate the true B cell ID phenomenon, leading to enhanced model training and improved predictive accuracy.

There are other limitations regarding the gap between computational biology and experimental biology. While our statistical analysis shows correlation of immunodominance, these findings do not necessarily indicate biological causation. Therefore, further experimental validation is required before these results can be translated into biological application. Additionally, some of the characteristics identified as significant, such as RSA and Protrusion in patch level, are correlated with each other, drawing attention to the result in terms of redundancy. (**Supplementary Figure S3**) While our model generalizes well to several types of antigens including virus, parasite, and human proteins, (**Supplementary Table S10**) the model has a limitation of not distinguishing neutralizing epitopes from non-neutralizing epitopes. However, it is reasonable to expect that the epitopes with high ID scores imply the neutralizing epitopes. To our knowledge, no prediction tools have attempted to predict direct neutralization. Future direction should aim for predicting the neutralizing effect to bridge the gap between physical interaction and biological function to enhance translational relevance.

## Data Availability

The datasets presented in this study can be found in online repositories. The names of the repository/repositories and accession number(s) can be found in the article/**Supplementary Material**. Code and data are available at https://github.com/sj584/BIDpred and webserver is available at https://bidpred.kaist.ac.kr/.
