# Supplementary material for: BIDpred: unraveling B cell Immunodominance hierarchical pattern using statistical feature discovery and deep learning prediction
Source: Front Immunol. 2025 Aug 13;16:1646946. doi: 10.3389/fimmu.2025.1646946 (PMC12380532; doi:10.3389/fimmu.2025.1646946)
Supplement: Supplementary file 1 [file Table1.docx]

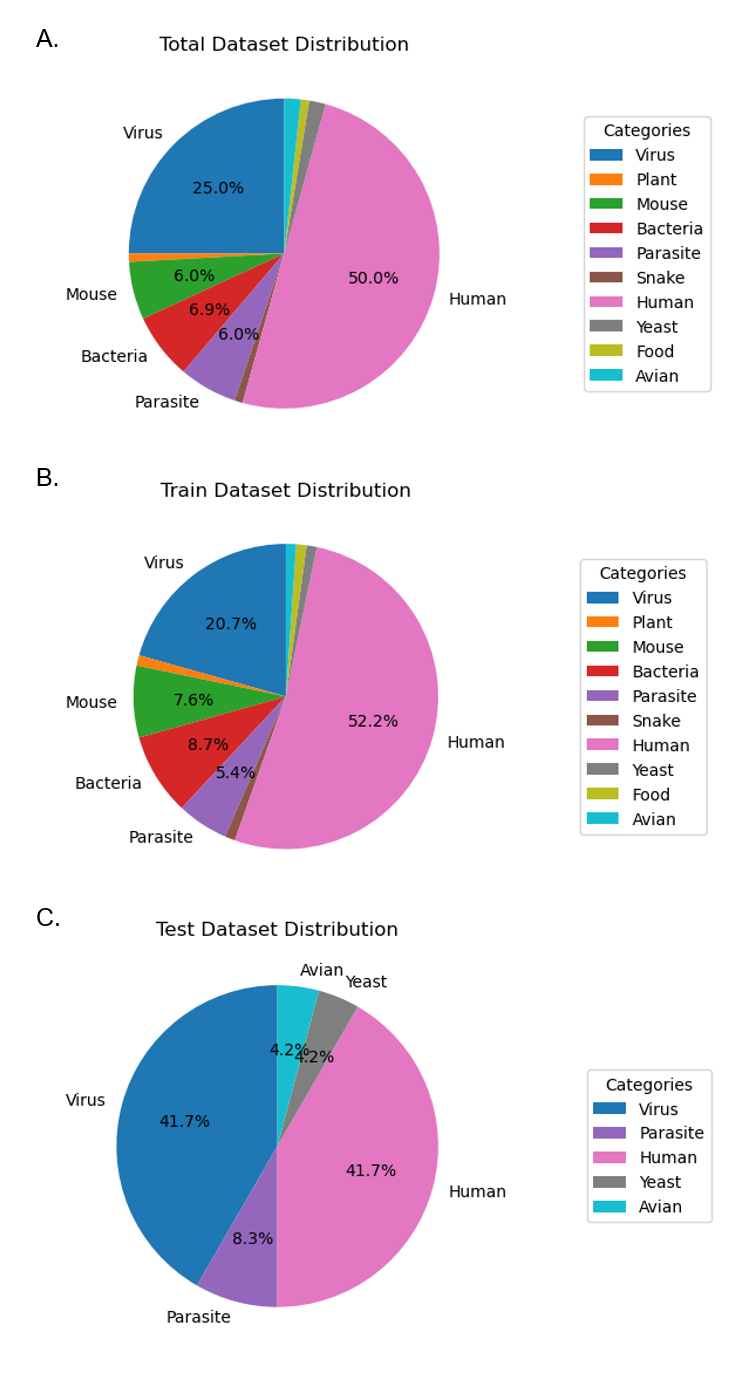


Supplementary Figure 1. Pie chart of each dataset shows antigen type distribution (A) Total dataset (B) Train set (C) Test set


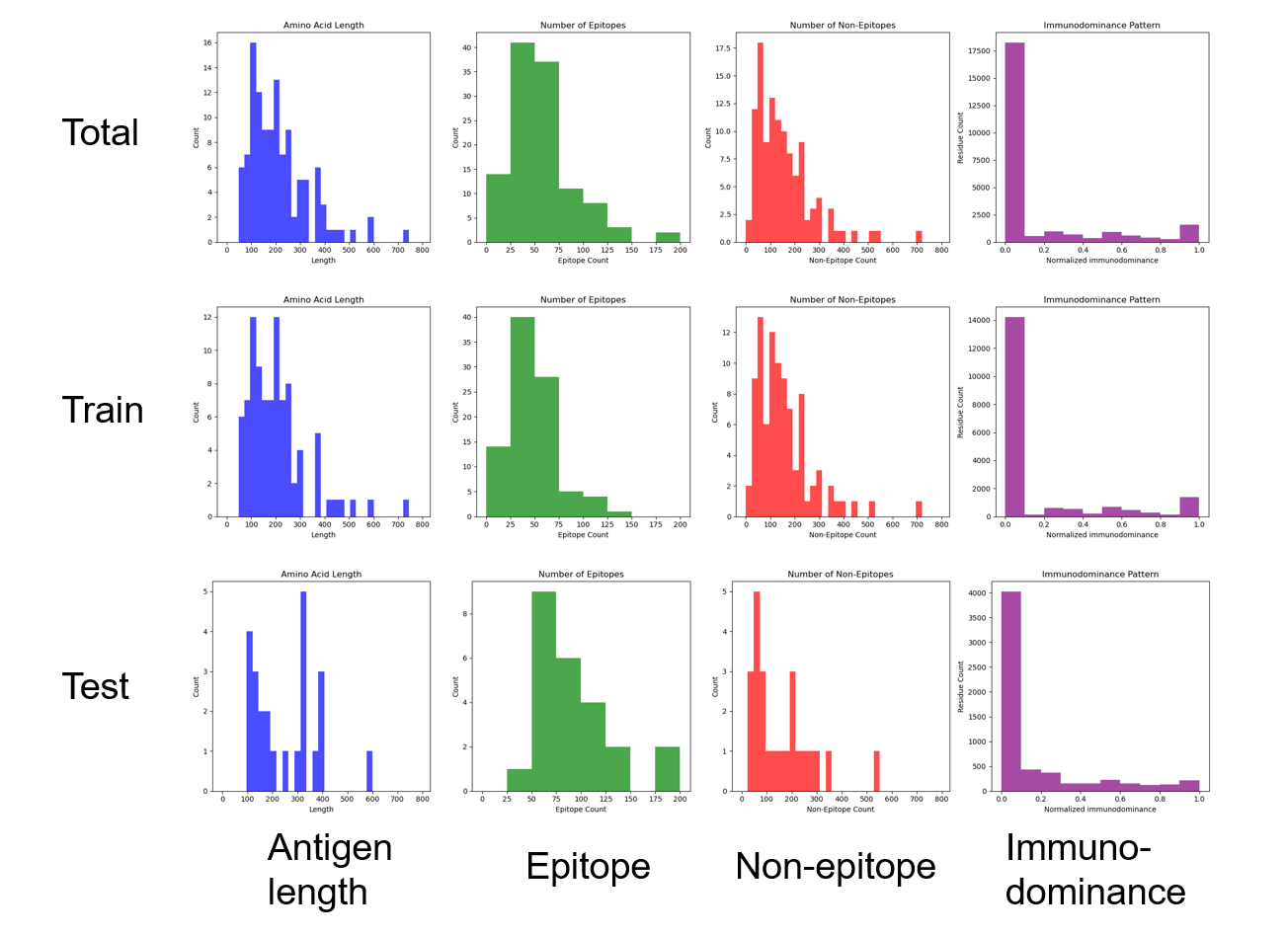


Supplementary Figure 2. Distribution of antigen size, epitope, non-epitope, immunodominance along the Total dataset, train set, and test set


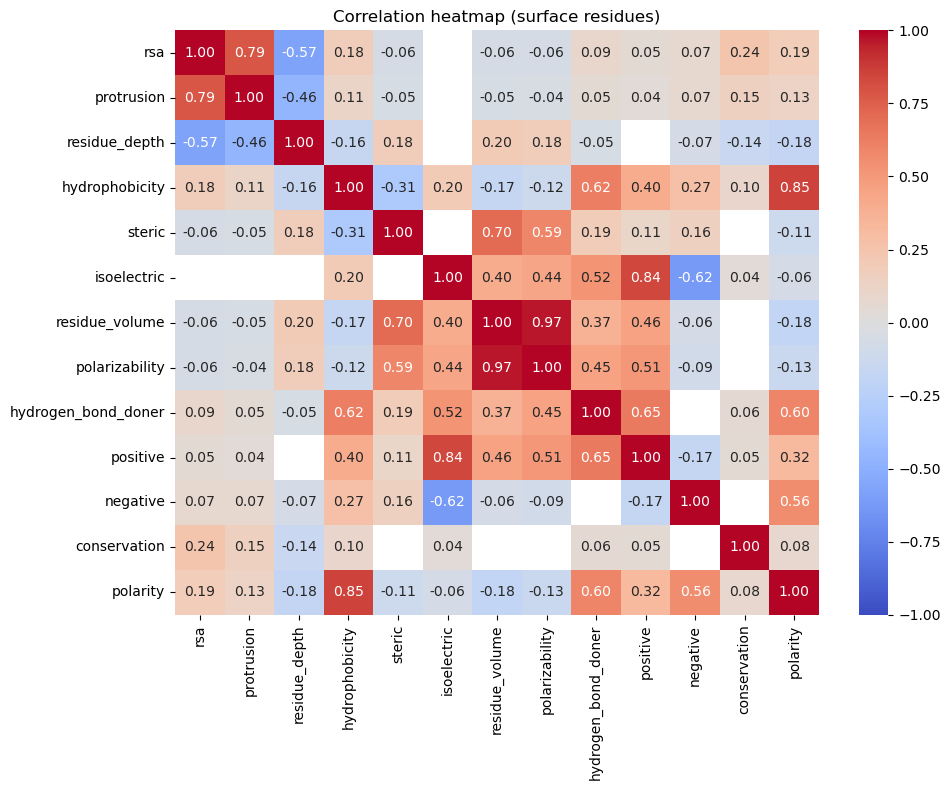


Supplementary Figure 3. A heatmap of the Pearson correlation coefficients between feature pairs. Statistically insignificant correlations, as determined by the Benjamini-Hochberg procedure, are indicated by empty (white) boxes.

**Supplementary Table 1** MSA depth of each cluster in the dataset

| MSA depth of cluster | Count |
| --- | --- |
| 271 | 1 |
| 48 | 1 |
| 25 | 1 |
| 23 | 1 |
| 20 | 1 |
| 19 | 1 |
| 18 | 1 |
| 17 | 2 |
| 14 | 1 |
| 13 | 1 |
| 12 | 7 |
| 11 | 3 |
| 10 | 3 |
| 9 | 2 |
| 8 | 8 |
| 7 | 5 |
| 6 | 17 |
| 5 | 17 |
| 4 | 43 |


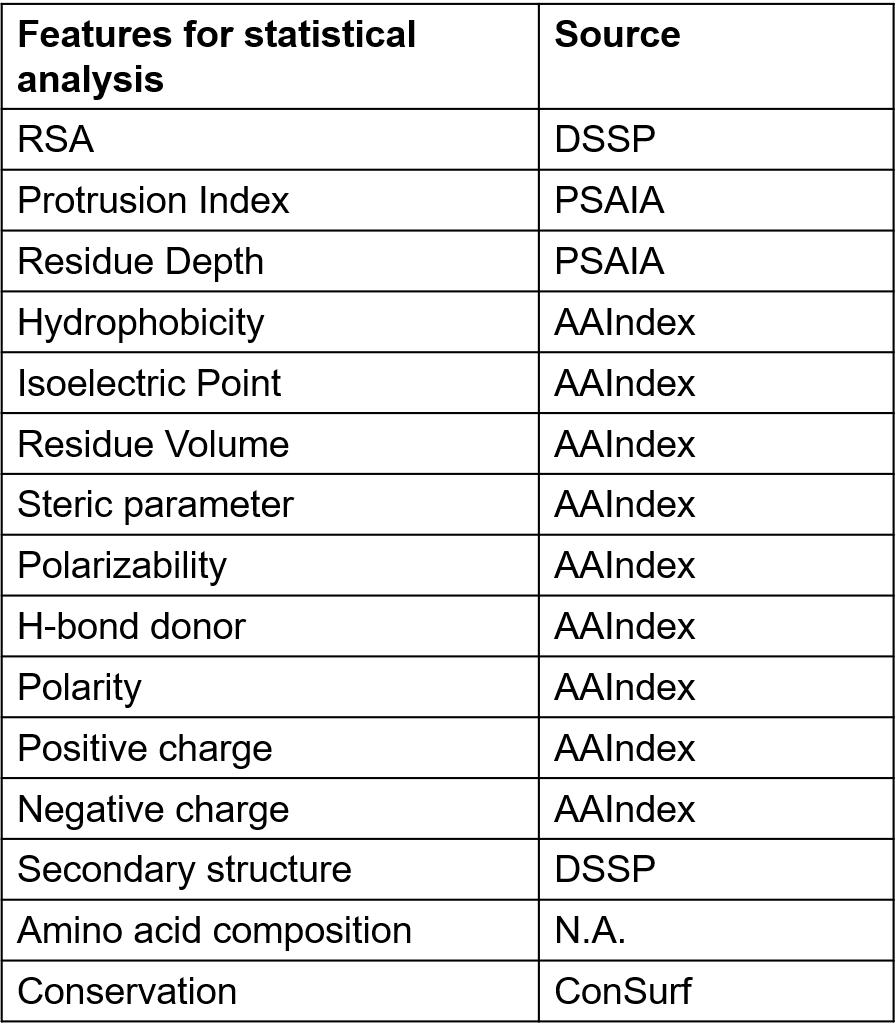


Supplementary Table 2 Features used for statistical analysis and the sources


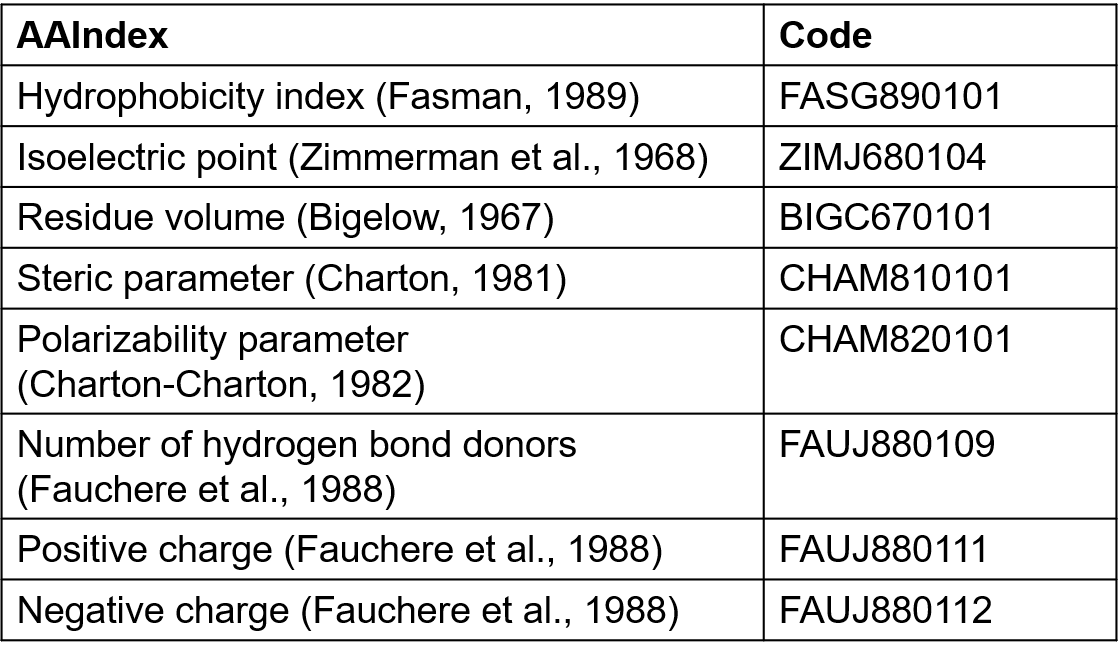


Supplementary Table 3 AAIndex description and codes

Supplementary Table 4 Details of features which showed significance in residue-level and patch-level statistical analysis


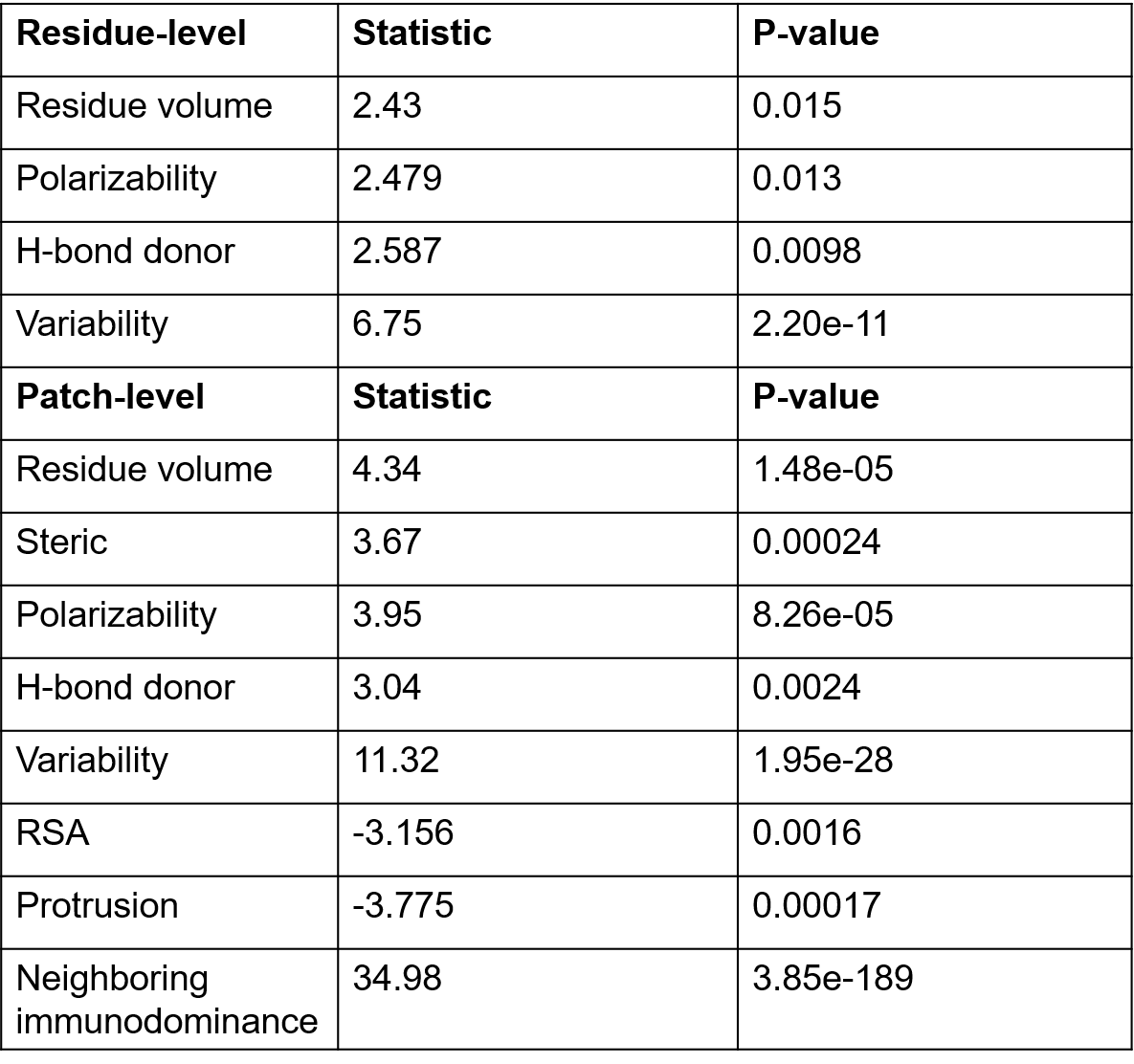


Supplementary Table 5 P-value details in ID benchmark test


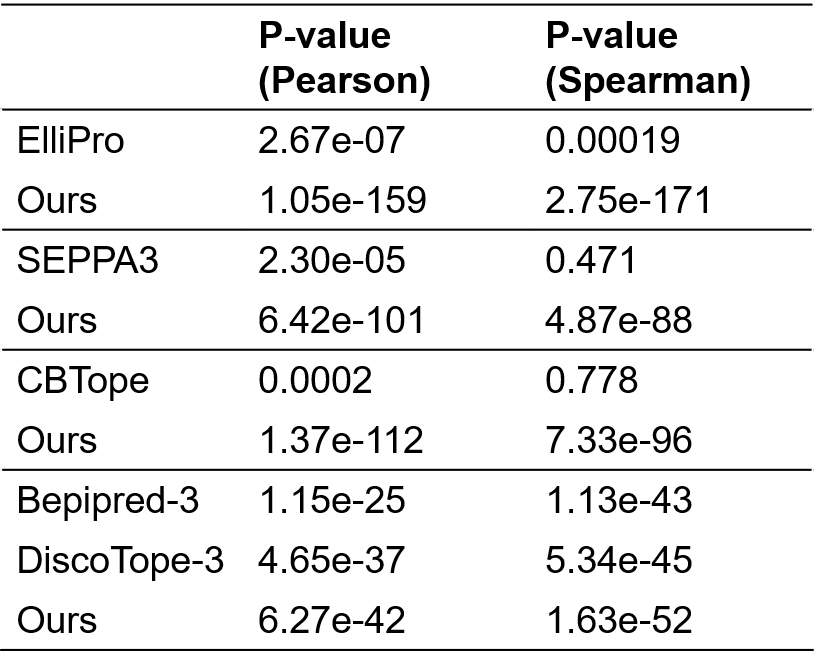


Supplementary Table 6 Result of all models evaluated on the same independent test set. Test set was uniformly reduced with 70% sequence similarity considering each model training set. (4 PDBs).


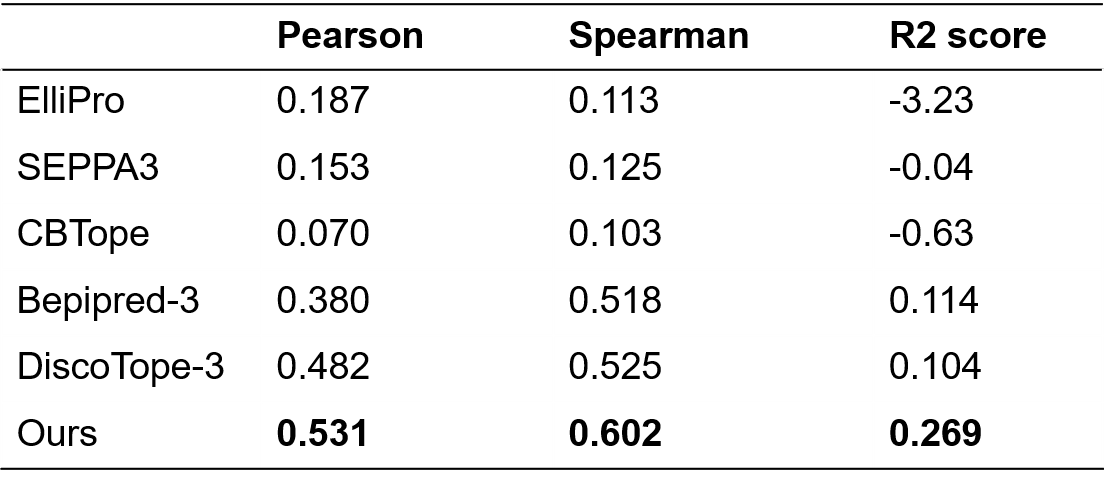


Supplementary Table 7 P-value details in Ablation study


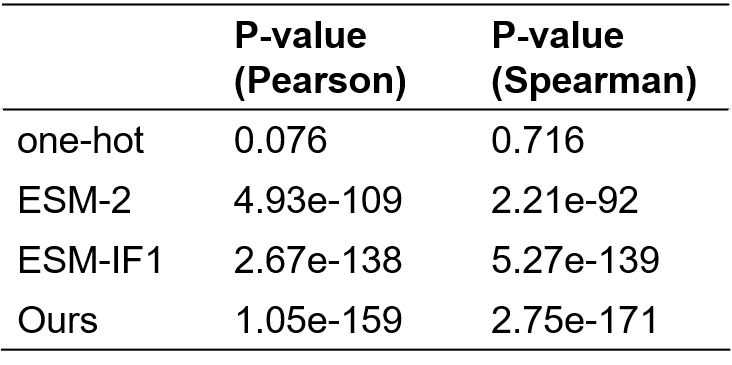


**Supplementary Table 8** SARS-CoV-2 Case Study results in comparison with other tools


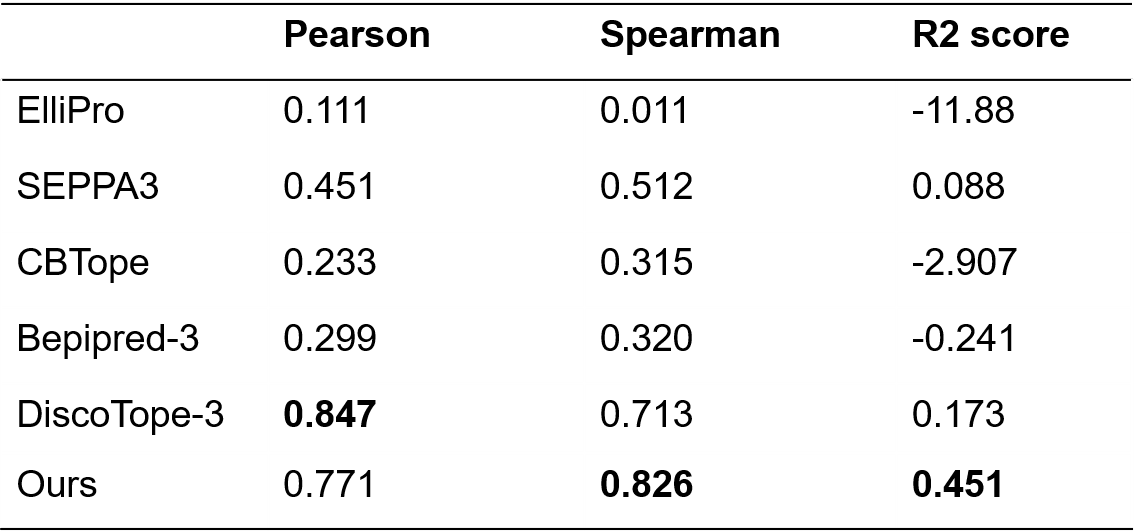


Supplementary Table 9. Evaluation result of random-split train/test dataset and half-split train/test result. Ratio means Number of proteins with MSA depth $\boldsymbol{\geq}$ 10 dividied by the number of dataset. (#) means number of proteins with MSA depth $\boldsymbol{\geq}$ 10 in the dataset.


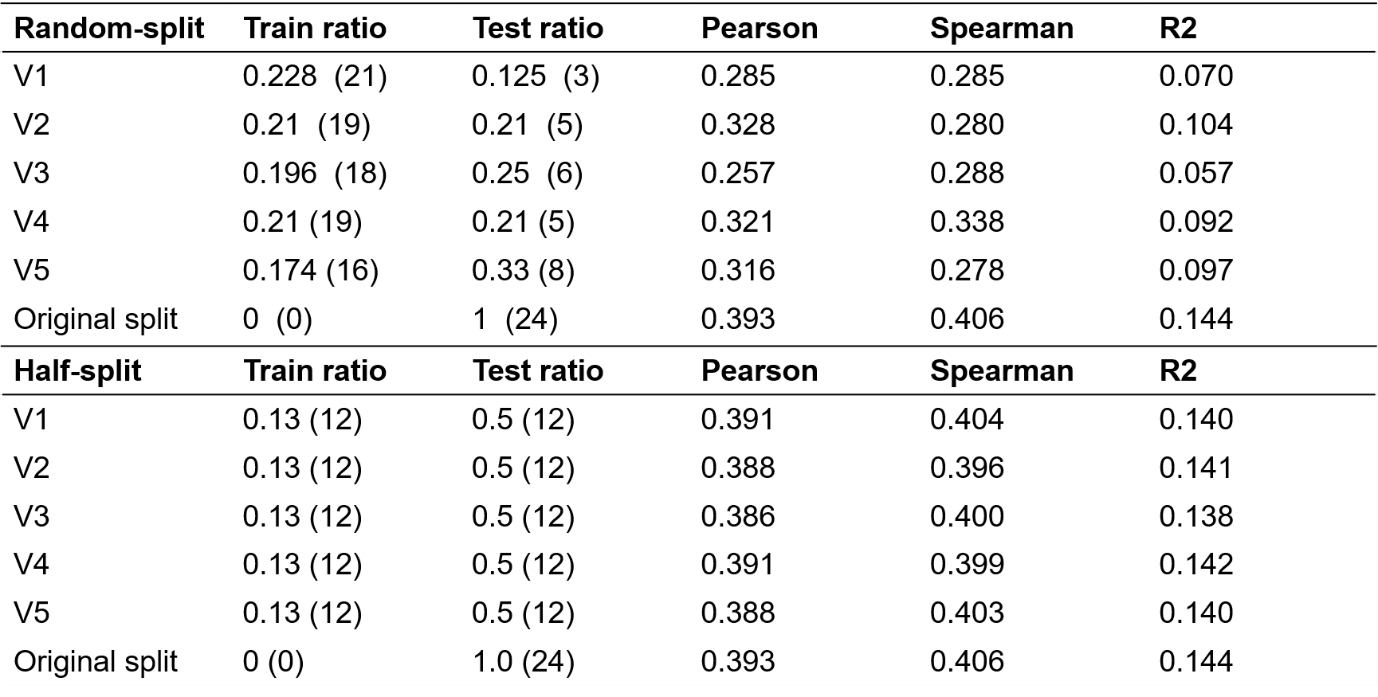


Supplementary Table 10. Test set cases which show good model generalization


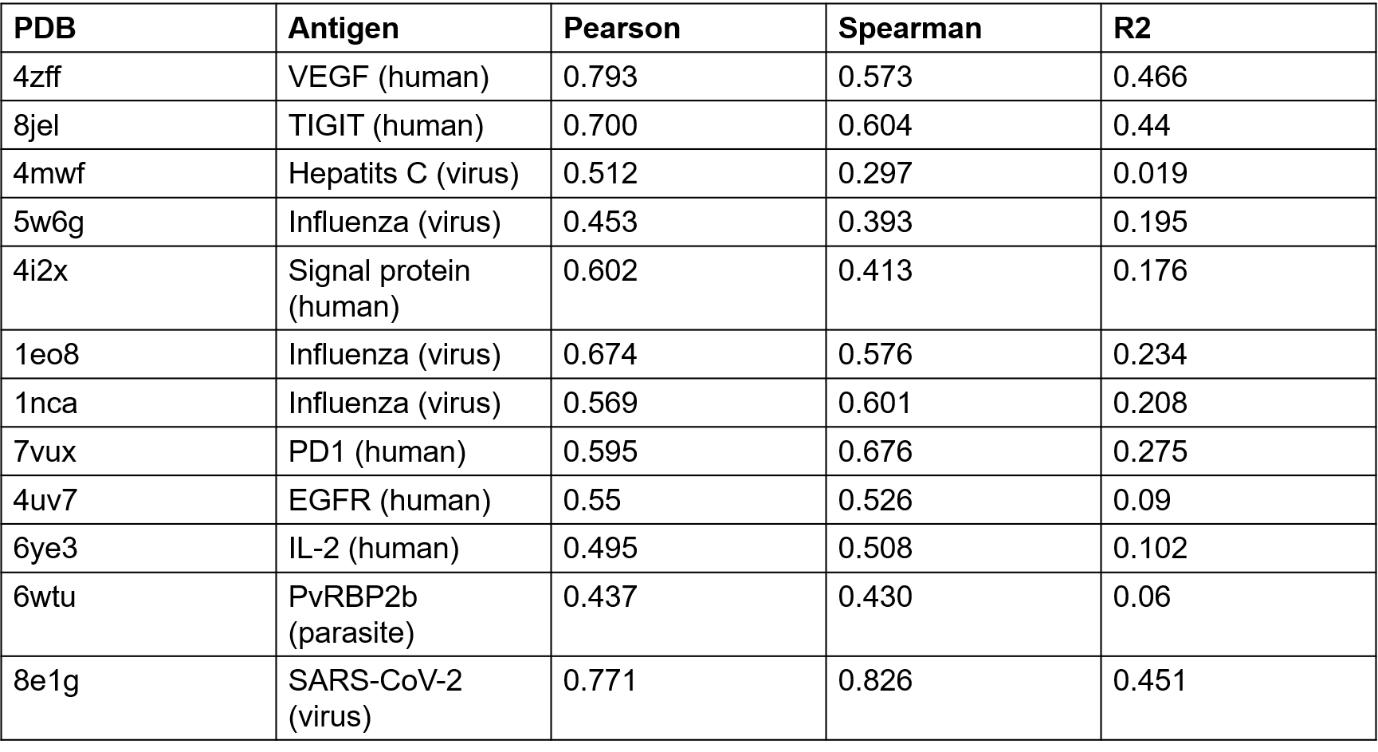


**Supplementary Table 11.** Antigen type of dataset

| PDB ID | Antigen source | Description |
| --- | --- | --- |
| 1eo8 | Virus | Influenza virus hemagglutinin |
| 4zff | Human | VEGF |
| 8ee8 | Virus | ZIKV E glycoprotein |
| 7vux | Human | PD1 |
| 5a3i | Virus | Influenza A H5 haemagglutinin |
| 4xak | Virus | MERS Co-V RBD |
| 4i2x | Human | Signal-regulatory protein gamma |
| 8jel | Human | TIGIT (T-cell immunoreceptor with Ig and ITIM domains) |
| 6wtu | Parasite | Plasmodium vivax reticulocyte binding protein 2b (PvRBP2b) |
| 1nca | Virus | THE INFLUENZA VIRUS N9 NEURAMINIDASE |
| 4uv7 | Human | Extracellular domain of EGFR |
| 4mwf | Virus | Structure of Hepatitis C Virus Envelope Glycoprotein E2 core |
| 8gpu | Virus | Yellow fever virus |
| 4rrp | Yeast | antigen Asf1p |
| 4qhu | Human | IL-17A |
| 5d72 | Human | Granulocyte-macrophage colony-stimulating factor |
| 4ypg | Human | Human Interferon alpha-2 |
| 6ye3 | Human | IL-2 |
| 8e1g | Virus | SARS-CoV-2 RBD |
| 3jwd | Virus | HIV-1 gp120 envelope glycoprotein |
| 5w6g | Virus | Influenza hemagglutinin H1 Solomon Islands |
| 4j4p | Human | Human ig epsilon chain C region |
| 1fbi | Avian | Guinea Fowl Lysozyme |
| 6phb | Parasite | Pfs25 |
| 5yoy | Human | Human tumor necrosis factor |
| 7s7i | Human | MICA alpha3 domain |
| 5tud | Human | Extracellular Recognition of human serotonin 2B receptor |
| 7rxl | Parasite | C-terminal alpha-TSR domain of P.falciparum |
| 5ush | Virus | Vaccinia virus D8 protein |
| 3pnw | Human | human TDRD3 |
| 7uvi | Parasite | Pfs230 domain 1. Plasmodium falciparum |
| 7pa9 | Virus | JC pollyomavirus VP1 |
| 6mtn | Virus | HIV-1 BG505 SOSIP.664 Prefusion Env Trimer Bound to Small Molecule HIV-1 Entry Inhibitor Compound |
| 6iek | Virus | RVFV Gn |
| 6umx | Human | Growth/differentiation factor 8 |
| 8db4 | Food | Peanut allergen Ara h 2 |
| 4rgm | Bacteria | Staphylococcal Enterotoxin B |
| 7x8t | Human | Frizzled 10 CRD |
| 7s13 | Mouse | Mouse CD96 dimer |
| 6hf1 | Mouse | Mutant oxidoreductase fragment of mouse QSOX1 |
| 6vmj | Human | Human Complement Factor D |
| 6mi2 | Human | Human 4-1BB |
| 6xm2 | Human | Human TGFb2 |
| 7n3c | Virus | N-terminal Domain of Nucleocapsid protein from SARS CoV-2 |
| 7ket | Bacteria | Meningococcal Factor H binding protein |
| 5mhr | Virus | T3D reovirus sigma1 |
| 7ly3 | Virus | SARS-CoV-2 S NTD |
| 6kyz | Virus | HRV14 3C |
| 6svl | Human | Human Myeloid-derived growth factor (MYDGF) |
| 5xj4 | Human | PD-L1 |
| 6o9i | Mouse | Ternary complex of mouse ECD |
| 6o3b | Human | Crystal structure of Frizzled 7 CRD |
| 3grw | Human | FGFR3 |
| 6mej | Virus | Hepatitis C virus envelope glycoprotein E2 ectodomain |
| 7bbj | Human | CD73 |
| 6mto | Virus | T117-F MPER scaffold |
| 6h2y | Bacteria | Neisseria meningitidis serogroup B |
| 4y5y | Human | Erythropoietin receptor |
| 1uj3 | Human | Tissue factor |
| 7xy8 | Human | CD147(EMMPIRIN) |
| 1osp | Bacteria | OUTER SURFACE PROTEIN A OF BORRELIA BURGDORFERI |
| 8ds5 | Human | CD27 |
| 7ox3 | Human | IL-9 |
| 4rdq | Avian | Calcium-activated chloride channel bestrophin-1 |
| 6ba5 | Human | Inactive tyrosine-protein kinase transmembrane receptor ROR1 |
| 7chz | Human | IL-1 beta |
| 4cad | Mouse | farnesylated CAAX protein processing by the integral membrane protease Rce1 |
| 7wg3 | Human | IL-17B receptor |
| 2adf | Human | von Willebrand factor A3-Domain |
| 1mhp | Mouse | chimeric alpha1 integrin I-domain |
| 6elu | Parasite | Serum Resistance Associated protein |
| 5wk3 | Human | CCL17 |
| 7qu2 | Virus | Junin virus GP1 glycoprotein |
| 3qa3 | Human | A-domain, integrin |
| 6vep | Human | Insulin receptor subunit alpha |
| 2j6e | Human | IG GAMMA-1 CHAIN C REGION |
| 4plj | Virus | Hepatitis E Virus E2s domain (Genotype IV) |
| 6j5f | Virus | tick-borne encephalitis virus envelope protein Domain III |
| 6nnf | Virus | HIV-1 BG505 SOSIP.664 Prefusion Env Trimer |
| 4dkf | Human | Interleukin-34 |
| 8jnk | Human | human ALKBH3 |
| 5vta | Mouse | DPPIV |
| 8d9y | Snake | Taipan alpha-neurotoxin |
| 5vlp | Human | PCSK9 |
| 4hc1 | Human | human MAdCAM-1 D1D2 |
| 6xlq | Human | Human BTN3A1 Ectodomain |
| 7lja | Human | Human TRAAK K+ channel FHEIG mutant A198E |
| 6u9s | Human | human CD81 large extracellular loop |
| 5cbe | Human | CXCL13 |
| 7yv1 | Human | Human K-Ras G12D (GDP-bound) |
| 4ywg | Virus | Scaffold HIV-1 gp120 V1V2 region derived from strain ZM109 |
| 3hi6 | Human | Integrin alpha-L |
| 3bn9 | Human | Membrane-type serine protease 1 |
| 8dfh | Parasite | Merozoite surface protein 1 |
| 4xvu | Yeast | ATPase GET3 |
| 8dn6 | Plant | Arabidopsis thaliana Toc75 POTRA domains |
| 6i8s | Human | Plasminogen activator inhibitor 1 |
| 7uxl | Parasite | malaria transmission-blocking antigen Pfs48/45-6C variant |
| 6ewb | Virus | GII.4 UNSW 2012 P domain |
| 5bjz | Bacteria | Maltose-binding protein |
| 5y9j | Human | BAFF |
| 6xsw | Human | Notch3 NRR |
| 7xjf | Human | Leukocyte surface antigen CD47 |
| 6cmg | Virus | Hendra Virus Attachment G Glycoprotein |
| 7a3o | Virus | dengue 1 virus envelope glycoprotein |
| 4i77 | Human | Interleukin-13 |
| 4h88 | Mouse | mouse PrPc Fragment 120-230 |
| 6vvu | Human | Tryptase alpha/beta-1 |
| 6yio | Human | CD25 ECD |
| 7lfa | Human | ApoL1 NTD |
| 6w7s | Bacteria | Ketoreductase from module 1 of the 6-deoxyerythronolide B synthase (KR1) |
| 3nh7 | Human | BMP type I receptor IA |
| 5ggv | Human | CTLA-4 |
| 2xra | Virus | gp41 mimetic 5- Helix |
| 6cyf | Bacteria | PcrV fragment |
| 1ymh | Bacteria | protein L (PpL) mutant A66W |
